# Modeling of Stress Relaxation Behavior in HDPE and PP Using Fractional Derivatives

**DOI:** 10.3390/polym17040453

**Published:** 2025-02-09

**Authors:** Karla L. Segura-Méndez, Jesús G. Puente-Córdova, Flor Y. Rentería-Baltiérrez, Juan F. Luna-Martínez, Nasser Mohamed-Noriega

**Affiliations:** 1 Facultad de Ingeniería Mecánica y Eléctrica, Universidad Autónoma de Nuevo León, Av. Universidad s/n, Cd. Universitaria, San Nicolás de los Garza 66455, Mexico; karla.seguramz@uanl.edu.mx (K.L.S.-M.); juan.lunamrt@uanl.edu.mx (J.F.L.-M.); nasser.mohamednr@uanl.edu.mx (N.M.-N.); 2 Facultad de Ciencias Químicas, Universidad Autónoma de Nuevo León, Av. Universidad s/n, Cd. Universitaria, San Nicolás de los Garza 66455, Mexico; flor.renteriabltz@uanl.edu.mx

**Keywords:** fractional calculus, stress relaxation, viscoelasticity, polypropylene, polyethylene

## Abstract

In this work, the viscoelastic behavior of high-density polyethylene (HDPE) and polypropylene (PP) was studied through stress relaxation experiments conducted at different strain levels. The main objective was to evaluate classical, fractional, and conformable derivatives to analyze molecular mobility, using statistical methods to identify the most accurate representation of the viscoelastic response. Besides the coefficient of determination (R^2^), the average absolute deviation (*AAD*) and mean squared error (*MSE*) were used as evaluation metrics, along with a multivariate analysis of variance (MANOVA) and the response surface methodology (RSM) to optimize the correspondence between experimental data and model predictions. The findings demonstrate that the spring-pot, Fractional Maxwell (FMM), Fractional Voigt–Kelvin (FVKM), and Kohlrausch–Williams-Watts (KWW) models effectively describe stress relaxation under statistical criteria. However, a joint analysis using RSM revealed that the choice of mathematical model significantly influences the outcomes. The FVKM was identified as the most effective for HDPE, while the KWW model best characterized PP. These results highlight the importance of optimization tools in advancing the characterization of polymer viscoelasticity. The ability to select the most accurate models for HDPE and PP under varying conditions can directly improve the performance and durability of products in critical industrial sectors such as packaging, automotive, and medical devices, where long-term mechanical behavior is crucial. By offering a framework adaptable to other materials and modeling approaches, this work provides valuable insights for optimizing polymer processing, improving product design, and enhancing the reliability of polymer-based components in a range of industrial applications.

## 1. Introduction

Polyolefins are synthetic polymers that have an extensive market at an industrial level. Today, many applications derived from the mechanical point of view are associated with those where structural, lightweight support and vibration-damping capacity are required. Polypropylene (PP) and polyethylene (PE) are two polyolefins that represent approximately 50% of global production and have a range of industrial applications, from packaging to automotive and aeronautical components [1,2]. The mechanical properties of PP and PE play an important role in the performance and suitability of their respective applications. The design and development of new polymeric materials require the study of viscoelastic behavior and relaxation phenomena. To follow the dynamics of relaxation phenomena, macroscopic properties such as mechanical, electrical, and thermal are monitored in time (frequency) or temperature domains. The evolution of these properties gives us information about specific molecular mobility and enhances our understanding of the structure–property relationship for polymers. The most frequently used experimental techniques for this purpose are dynamic mechanical analysis (DMA), dynamic electrical analysis (DEA), thermally stimulated discharge currents (TSDCs), and nuclear magnetic resonance (NMR) [3,4,5,6]. Although such techniques can provide valuable information about relaxation phenomena, polymer dynamics are complex and possess memory effects, often requiring the use of mathematical models to interpret experimental data.

From the rheological point of view, creep, stress relaxation, and DMA are the principal tests used to study the molecular mobility of polymeric materials [7,8,9]. Thus, the analysis of data obtained from rheological tests can be carried out through analogous mechanical models based on springs and dashpots. Constitutive equations are obtained to describe the stress–strain relationships, and differential equations of integer order can be used with the classical models of Maxwell, Voigt–Kelvin, Zener, and Burgers [10,11,12,13]. This generates significant deviations between theoretical predictions and experimental results, giving rise to errors reflected in the design of products and components. An alternative is using generalized models (parallel or series associations) to minimize the error, providing a better description of experimental results. Although this approach is commonly used in numerical simulation software, these programs can generate many parameters with complex physical interpretation. For this purpose, the use of fractional derivatives is justified as an innovative tool.

Fractional calculus is a powerful mathematical tool since it extends the integer order of derivatives and integrals to arbitrary orders (fractions, irrational, and complex numbers) [14,15]. This tool provides a deeper description of materials behavior, particularly in cases involving anomalous relaxation, time-dependent phenomena, and fractal structures. The fractional derivative is considered a nonlocal operator and has advantages related to describing complex systems with memory and hereditary properties. Several definitions are proposed to describe physical systems, including Riemann–Liouville, Caputo, Weyl, Grünwald–Letnikov, Caputo-Fabrizio, and Atangana–Baleanu [16,17,18]. However, due to the lack of a clear physical and geometric interpretation of the fractional derivative, some sectors of the scientific community do not accept it well enough.

Several authors have worked on this subject; for example, Podlubny developed an approach suggesting a physical interpretation of fractional integration and fractional differentiation based on the use of cosmic and individual times [19]. Moshrefi-Torbati and Hammond have shown that the order of a fractional derivative represents the rate at which some energy has been lost in a system, while the order of a fractional integral indicates the amount of energy still present or preserved [20]. Du et al. demonstrated that the physical meaning of fractional order can serve as an index of memory for describing memory phenomena [21]. More recently, Ruby and Mandal presented an interesting physical interpretation of fractional derivatives in terms of fractional divergence [22]. Regarding polymer viscoelasticity, Reyes-Melo et al. and Rentería-Baltiérrez et al. established a close relationship between fractional order and molecular mobility for mechanical and electrical relaxation phenomena [3,4,15,23,24,25].

There are several reports on modeling viscoelastic behavior in thermoplastics and thermosets. However, choosing the optimal model remains a challenge as the accuracy of predictions can vary significantly depending on deformation and temperature conditions. To address this issue, the use of advanced statistical methods, such as multivariate analysis of variance (MANOVA) and response surface methodology (RSM), has become increasingly widespread. For instance, Mameri et al. [26] optimized the preparation of HDPE specimens using RSM, minimizing damage during machining and improving mechanical property characterization. Their study highlighted significant differences in tensile properties between pipe layers, attributed to variations in crystallinity. Moreover, MANOVA has been used to analyze the effects of multiple factors on the mechanical and viscoelastic properties of polymers, helping identify the most influential parameters under varying conditions [27]. These techniques enable a more robust optimization of the correlation between experimental data and model predictions, allowing the evaluation of model accuracy through metrics like the coefficient of determination (*R^2^*), mean squared error (*MSE*), and average absolute deviation (*AAD*) [28]. Furthermore, statistical analysis aids in identifying the most suitable model for different operational conditions, which is crucial for tailoring polymer performance to specific industrial applications [29]. Despite these advancements, there is still limited consensus on the most effective methodology for characterizing materials such as HDPE and PP across a broad range of conditions. This work aims to fill this gap by comparing several mathematical approaches and leveraging statistical tools to optimize model selection, offering a more accurate and reliable characterization of polymer viscoelasticity. In this sense, we evaluate the viscoelastic behavior of two commercial polyolefins, HDPE and PP, through stress relaxation experiments under different strain steps. The principal objective is to compare three derivative definitions—classical, fractional, and conformable—to analyze viscoelastic models and employ statistical analysis to describe the best viscoelastic response. The following summarizes the rest of this paper: Section 2 introduces the classical and fractional viscoelastic models for modeling stress relaxation; Section 3 presents the experimental methodology; Section 4 presents the results and discussions about the modeling of stress relaxation; and finally, Section 5 reports the conclusions.

## 2. Fractional Viscoelastic Models

From a mechanical point of view, viscoelasticity represents an intermediate behavior between an ideal elastic solid and a pure viscous liquid, manifested in materials such as polymers, rubbers, fibers, tissues, and cells, among others [30,31,32,33]. Regarding polymer behavior, this results in properties that are a function of time due to their complex structure and morphology. The polymeric structure formed by long chains consists of a large number of repetitive units where covalent bonds prevail. Due to predominant bonding and inter- and intra-molecular interactions, structural conformations and rearrangements occur, manifesting in different types of molecular mobility: local motions and rotations of chemical groups, side groups and segmental motion, and chain slippage [4,34,35,36]. Polymers exhibit a characteristic morphology, which typically results in semicrystalline or amorphous states. A semicrystalline polymer consists of an intimate mixture of amorphous and crystalline phases. The amorphous phase lacks long-range order and is far from thermodynamic equilibrium, resulting in relaxation phenomena [24,37]. When these two phases are subjected to a mechanical stimulus, they exhibit two different mechanical responses with a characteristic relaxation time, producing complex viscoelastic behavior. This behavior can be analyzed using mechanical tests such as creep, stress relaxation, and DMA.

The creep test involves monitoring the strain ε over time t when a constant stress σ acts on the material, while the stress relaxation test consists of monitoring the stress σ over time t when a constant strain ε acts on the material. The compliance function $J\left(t\right)$ is used to analyze the response of the creep experiment, while the relaxation function E(t) is employed to study the stress relaxation response. Through hereditary integrals, shown in Equations (1) and (2), the theory of linear viscoelasticity predicts that it is possible to establish a relationship between the functions J(t) and E(t), which are not inversely proportional.

(1) $$\sigma \left(t\right)=\int_{0}^{t}E(t-t^{\prime })\frac{d\varepsilon \left(t^{\prime }\right)}{dt^{\prime }}dt^{\prime }$$

(2) $$\varepsilon \left(t\right)=\int_{0}^{t}J(t-t^{\prime })\frac{d\sigma \left(t^{\prime }\right)}{dt^{\prime }}dt^{\prime }$$

As previously mentioned, creep and stress relaxation tests are used to characterize viscoelastic behavior in the time domain. However, in most cases, long-term measurements are required to obtain a complete viscoelastic response. For this reason, the temperature factor is introduced through the principle of time–temperature superposition, where it is possible to obtain information on a broad spectrum of time (frequency) and temperature to construct master curves [38,39,40]. A disadvantage of these techniques is the complexity of separating the elastic component from the viscous component, which leads to the use of analogous mechanical models based on springs and dashpots. An ideal elastic material is described by a Hookean spring, as shown in Equation (3):

(3) $$\sigma \left(t\right)=ED_{t}^{0}\varepsilon (t)$$

where E is the elastic modulus and $D_{t}^{0}\varepsilon (t)$ is a derivative of order zero. Conversely, an ideal viscous material is described by a Newtonian dashpot, as shown in Equation (4):

(4) $$\sigma \left(t\right)=\eta D_{t}^{1}\varepsilon \left(t\right)$$

**Figure 1:**
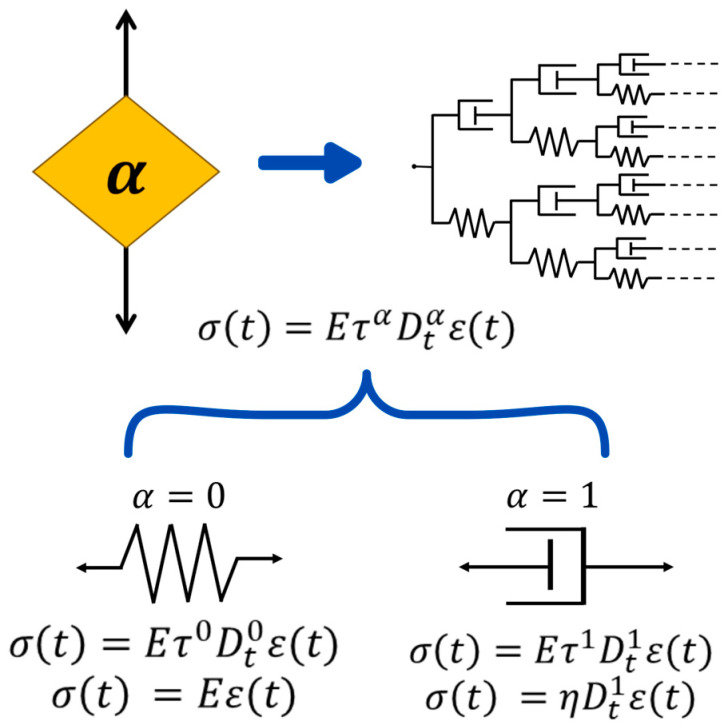
Spring-pot fractional element. When the fractional order α=0 the behavior corresponds to spring element (Hooke´s law), and when α=1 the behavior corresponds to dashpot element (Newton´s law).

In this equation, η is the viscosity of the material and $D_{t}^{1}\varepsilon \left(t\right)$ is the shear rate (strain time derivative). Equations (3) and (4) are linear and based on integer-order operators. Consequently, combining these two constitutive elements should represent the viscoelastic behavior of polymers. There is a common practice of combining springs and dashpots, resulting in differential equations that tend to explain the intermediate rheological behavior between an elastic solid and a viscous liquid. The Maxwell and Voigt–Kelvin models are used to describe the viscoelastic response of polymeric materials as a first approximation. The Maxwell model consists of a series arrangement of a spring and a dashpot, while the Voigt–Kelvin model consists of a parallel arrangement of a spring and a dashpot. This is based on the premise that both elements and properties manifest themselves on different time scales. To represent an intermediate behavior between elasticity and viscosity, Scott-Blair proposed the use of a new rheological element that interpolates between a solid and liquid behavior, which Koeller later named a spring-pot, as shown in Figure 1 [41,42,43].

Several authors have shown that the differential equation governing the spring-pot element (SP), as shown in Equation (5), can be physically obtained using hierarchical combinations of springs and dashpots [44,45,46].

(5) $$\sigma \left(t\right)=E\tau^{\alpha }D_{t}^{\alpha }\varepsilon \left(t\right)$$

The relaxation time is defined as τ=η/E, referring to the adjustment to new equilibrium conditions in the polymer when an external variable is modified. $D_{t}^{\alpha }\varepsilon \left(t\right)$ is the fractional derivative under the Caputo definition, as shown in Equation (6).

(6) $$D_{t}^{\alpha }\varepsilon \left(t\right)=\frac{1}{\Gamma \left(n-\alpha \right)}\int_{0}^{t}\frac{\varepsilon^{n}\left(\xi \right)}{{\left(t-\xi \right)}^{n+\alpha -1}}\mathrm{d}\xi$$

The Euler gamma function is represented by Γ(x), α is the fractional order that takes values between n−1<α≤n, ξ is an integration variable, and $\varepsilon^{n}$ is the derivative of order n. The Caputo derivative presents a power-law kernel. In this definition, the derivative of a constant is zero (unlike the Riemann–Liouville case). When used in fractional differential equations, the initial conditions correspond to what is physically measured, which is the reason to be applied in physics and engineering. For a fractional order α=0, the spring-pot behaves like a Hookean material, and if α=1, the element produces Newtonian behavior. In this sense, to obtain fractional models for solid viscoelastic materials, one strategy involves replacing the dashpot element with the spring-pot in classical models [37,47,48]. This results in a fractional differential equation that can be solved for creep and stress relaxation tests. To accomplish this, the Laplace transform must be applied to Equations (1) and (2), resulting in Equations (7) and (8).

(7) $$\bar{\sigma }\left(s\right)=s\;\bar{E}\left(s\right)\bar{\varepsilon }\left(s\right)$$

(8) $$\bar{\varepsilon }\left(s\right)=s\;\bar{J}\left(s\right)\bar{\sigma }\left(s\right)$$

**Figure 2:**
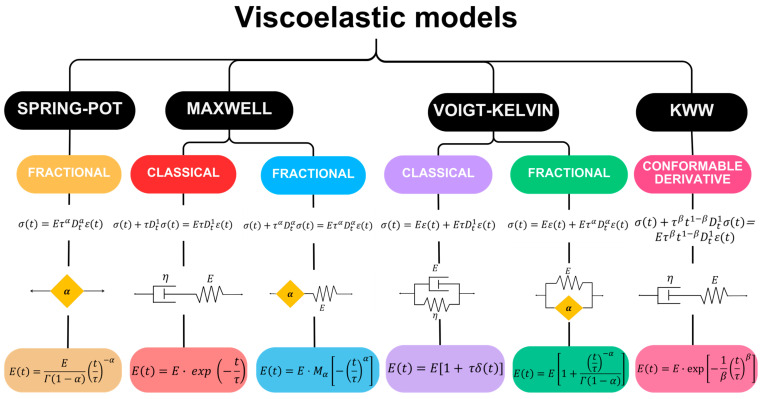
Scheme of viscoelastic models used for the analysis of experimental data. Each viscoelastic model is presented, with its respective differential equations, mechanical arrangements, and solutions for the relaxation function E(t). Γ(∙) is the gamma function, δ(t) is the Dirac-delta function, and $M_{\alpha }$ is the Mittag–Leffler function of one parameter.

This makes it possible to employ Laplace transforms to solve integer and fractional differential equations and then obtain the representations of the creep and relaxation functions. Figure 2 presents a scheme of the viscoelastic models used in this work [33,47,49]. The main models are spring-pot, Maxwell, and Voigt–Kelvin. These are presented according to the derivative definition type: classical, fractional, and conformable. The solution from differential equations (integer and fractional order) is also presented, but only for the relaxation modulus E(t).

Regarding the conformable derivative, Khalil proposed this concept in 2014 as a simple mathematical tool for solving fractional differential equations [50]. The conformable derivative of order β is defined as follows, for all x>0 and 0<β ≤ 1.

(9) $$D_{x}^{\beta }f\left(x\right)=\underset{h\to 0}{\lim}\;\frac{f\left(x+hx^{1-\beta }\right)-f\left(x\right)}{h}$$

In Equation (9), the operator $D_{x}^{\beta }$ can be considered a generalization of the definition established by traditional calculus for the first-order derivative of a function f(x), where β=1, and Equation (9) is transformed into Equation (10), which corresponds to the definition of the first-order derivative, $D_{x}^{1}f\left(x\right)=f^{\prime }(x)$.

(10) $$D_{x}^{1}f\left(x\right)=\underset{h\to 0}{\lim}\;\frac{f\left(x+h\right)-f\left(x\right)}{h}$$

In this sense, Puente-Córdova et al. [51] proposed its application in the fractional Maxwell model, obtaining a solution of type KWW (Kohlrausch–Williams–Watts) for the relaxation modulus. More recently, Kachhia and Gosai [52] solved the fractional cases of the Maxwell and Zener models for E(t), as well as the creep compliance function J(t) using the conformable derivative approach.

## 3. Materials and Methods

The commercial polyolefins used in this work are polypropylene (PP) and high-density polyethylene (HDPE), which were purchased from CYPMA, México. The samples were manufactured with a dog-bone shape Type I (ASTM D638) through the injection molding process. Structural characterization was carried out using Fourier transform infrared spectroscopy (FTIR) to corroborate the polymer structure. The spectrum was measured in transmittance mode at wavenumbers from 400 to 4000 cm^−1^ using a Perkin–Elmer Spectrum 100 spectrometer.

For structural applications, it is important to know the thermal stability of polymeric materials. In this sense, a thermogravimetric analysis (TGA) was carried out using SDT-Q600 TA Instruments equipment, employing a temperature range from 293 to 1073 K at a heating rate of 10 K/min and under a nitrogen atmosphere. Simultaneous differential scanning calorimetry (DSC) measurement was performed to determine the fraction of the crystalline phase. The dynamic mechanical analysis (DMA) was performed to measure the viscoelastic behavior of PP and HDPE as a function of temperature, using the complex elastic modulus $E^{*}=E^{\prime }+iE^{\prime \prime }$. In this way, a Perkin Elmer DMA8000 was used in tension mode, from a temperature range of 300 K to the flow temperature of each polymer at a frequency of 1 Hz, and a heating rate of 2 K/min. The dimensions of the samples were 1 mm × 3.5 mm × 10 mm.

Stress relaxation experiments were performed using polymer samples Type I (ASTM D638), using a Shimadzu AGS-X-10 kN universal testing machine. The accomplished experiments had multi-steps strain ε of 0.5, 1, 5, and 10%. The duration of each step was 1000 s, and three samples of PP and HDPE were used for each experiment.

Therefore, the data were adjusted using the curve-fitting toolbox from Matlab R2019a software. The analyzed curves obtained from the fitting used three statistical evaluators: *R*^2^ (coefficient of determination), *AAD* (average absolute deviation), and *MSE* (mean squared error).

(11) $$R^{2}=1-\frac{\sum_{i=1}^{N}{\left(y_{i}-y_{fit}\right)}^{2}}{\sum_{i=1}^{N}{\left(\bar{y}-y_{fit}\right)}^{2}}$$

(12) $$AAD=\frac{1}{N}\sum_{i=1}^{N}\left|\frac{y_{i}-y_{fit}}{y_{i}}\right|$$

(13) $$MSE=\frac{1}{N}\sum_{i=1}^{N}{\left(y_{i}-y_{fit}\right)}^{2}$$

where N is the number of data measured, $y_{i}$ is the experimental value, $y_{fit}$ is the value predicted by the model, and $\bar{y}$ is the average of the experimental data.

According to the statistical analysis, the tests were conducted using Minitab 19 statistical software, where the fits of six mathematical models were employed for analyzing both polymers. A significance level of 5% was applied, utilizing both multivariate analysis of variance (MANOVA) and the response surface methodology (RSM) to evaluate the performance and suitability of the models. Additionally, the selection of MANOVA was justified due to the significant correlation found among the three response variables, assessed using Pearson’s correlation test. Also, the RSM was chosen as the optimization technique due to the significant curvature observed in the regression results. This combination of methods allowed for a more robust analysis and a better understanding of the interactions between the studied variables.

## 4. Results and Discussion

### 4.1. Structural and Thermal Characterization

**Figure 3:**
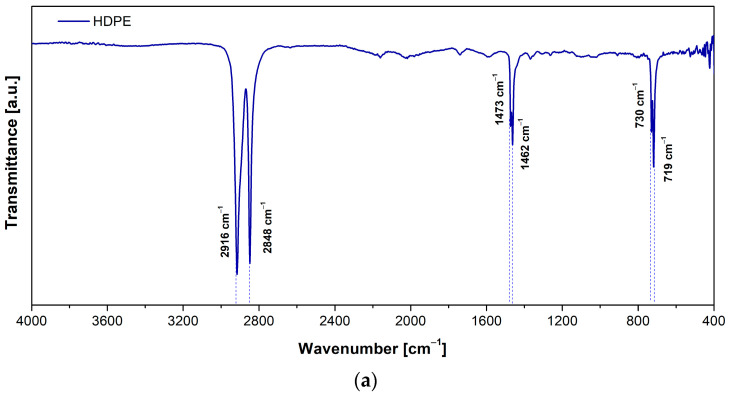

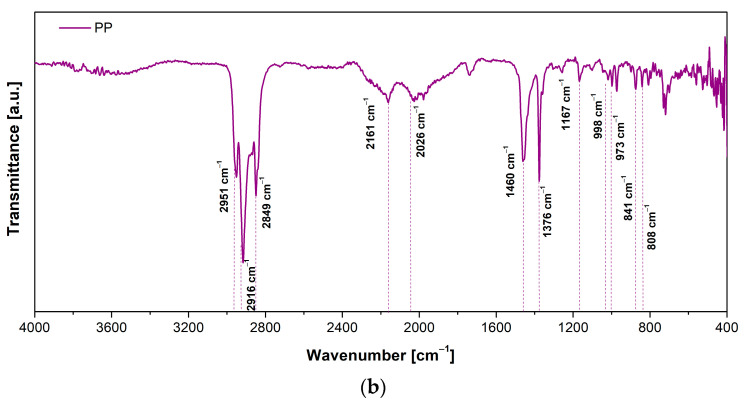
Infrared spectrum for (**a**) HDPE and (**b**) PP.

The FTIR analysis was conducted to confirm the polymer structures of the commercial HDPE and PP samples used in this study. The spectra confirm well-known assignments of the chemical groups present in these polymers, ensuring the reliability of the materials analyzed. FTIR spectra are shown in Figure 3a,b, where the vibration modes of chemical groups for HDPE and PP are identified. These spectra exhibit the characteristic absorption bands of polymer structures. Regarding HDPE, the positions found in the spectra are assigned as follows: 2916 and 2848 cm^−1^ correspond to the asymmetric and symmetric vibration of groups CH_2_, with relative intensities of 1.00 and 0.93, respectively. The bands at 1473 and 1462 cm^−1^ associated with CH_2_ bending deformation exhibit relative intensities of 0.35 and 0.43, respectively. Similarly, the bands at 730 and 719 cm^−1^, attributed to CH_2_ rocking deformation, have relative intensities of 0.38 and 0.53. According to the literature [53,54], these positions and relative intensities are consistent with the structure of HDPE. Moreover, absorption bands for PP are identified as follows: CH_3_ groups are found at 2951 cm^−1^ for asymmetrical stretching, with a relative intensity of 0.57, while 2916 cm^−1^ corresponds to the asymmetrical stretching of CH_2_, with a relative intensity of 1.00. The symmetrical stretching vibration of CH_3_ at 2849 cm^−1^ has a relative intensity of 0.66. The bending vibration modes of CH_2_ at 1460 and 1376 cm^−1^ show relative intensities of 0.49 and 0.59, respectively. The band at 1167 cm^−1^, attributed to C-C asymmetric stretching, has a relative intensity of 0.09, while the bands at 998 and 973 cm^−1^, due to CH_3_ asymmetric rocking vibration, show intensities of 0.11 and 0.14, respectively. The bands at 841 and 808 cm^−1^ are due to CH_2_ rocking vibrations, with intensities of 0.11 and 0.10, respectively. The bands at 2161 and 2026 cm^−1^, observed in the spectrum of polypropylene (PP), are attributed to additives or plasticizers typically present in commercial formulations. Similar bands have been reported in the literature, highlighting their characteristic presence in such materials without further discussion [55,56,57].

**Figure 4:**
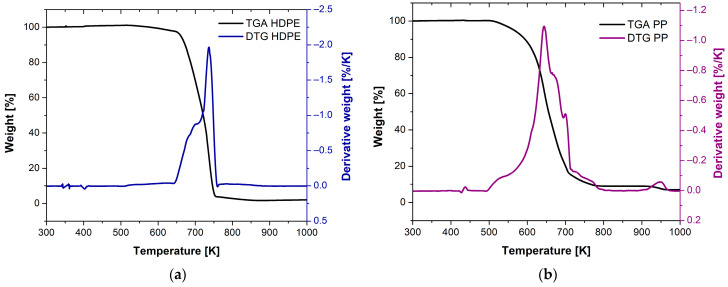
TGA/DTG results for (**a**) HDPE and (**b**) PP.

The thermogravimetric analysis (TGA) and derivative thermogravimetric (DTG) results for HDPE and PP indicate a predominant single-stage degradation process, as evidenced by the single peak observed in the DTG curves in Figure 4a,b. For HDPE, this degradation occurs in the temperature range from 617 to 773 K, with a maximum peak at 731 K [58], while for PP, the degradation range spans from 493 to 780 K, with a maximum peak at 644 K [59]. These thermal behaviors are associated with the breakdown of polymer chains under high temperatures. While the single peak observed in the DTG suggests a dominant degradation mechanism, it is important to note that real-world polymer systems, particularly commercial-grade materials, often contain additives or other components that may influence the thermal degradation process. Secondary degradation events may occur, although they may not be clearly resolved in the DTG due to their relatively low intensity or overlap with the main degradation peak.

While the TGA and DTG results for HDPE and PP indicate a predominant single-stage degradation process, the presence of additives or plasticizers, as suggested by the FTIR analysis, could influence the thermal degradation behavior. These components may act to stabilize or destabilize the polymer chains, potentially leading to secondary degradation events or slight modifications to the degradation profile that may not be fully resolved in the DTG curves. This highlights the complexity of commercial-grade polymer systems and the interplay between the polymer matrix and its additives during thermal degradation.

DSC was used as a simultaneous technique with TGA to calculate the degree of crystallinity for both polymers. The melting enthalpy of HDPE at 399 K was calculated to be 227.3 J/g. Considering the ideal enthalpy for 100% crystalline HDPE reported to be 293 J/g [60], the obtained crystallinity was ~77.5%. Some reports consider HDPE as a polymer with a maximum crystallinity of 80%. In the case of PP, the melting enthalpy at 428 K was calculated to be 41.6 J/g. The melting enthalpy for 100% crystalline PP is reported as 207 J/g [61], and a crystallinity of about ~20.1% was calculated. These results confirm the semicrystalline nature of HDPE and PP, which significantly influences their thermal and mechanical properties.

**Figure 5:**
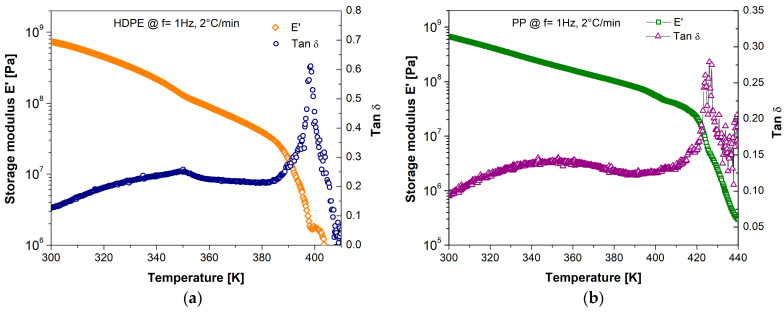
Storage modulus and Tan δ for (**a**) HDPE and (**b**) PP.

In Figure 5a,b, the DMA results for HDPE and PP are presented. For both polymers, there is a specific mechanical response of storage modulus E’ dependent on temperature. Semicrystalline polymers typically present three relaxation phenomena (γ, β, and α) in the temperature range of liquid N_2_ to the flow temperature (Tm). The γ process occurs at low temperatures and is generated in the amorphous phase, although it also has characteristics associated with the crystalline phase. The β process in semicrystalline polymers is typically associated with the glass transition, which is directly related to the amorphous phase. This has a notable difference in amorphous polymers (like PVC, PVB, or PMMA), where α relaxation is directly associated with the mechanical manifestation of the glass transition. The α process manifests itself at high temperatures (typically above room temperature), which represents a mechanical relaxation associated with the molecular mobility of the crystalline phase [62,63]. Identifying the relaxation processes associated with specific molecular motions will deepen our understanding of the structure–property relationships in polymers and their impact on the performance of products and components in service.

In this context, the manifestations of mechanical relaxation phenomena have been reported for HDPE and PP, with the obtained values showing consistency [62,64]. The γ and β processes are related to the molecular mobility of polymer chains in the amorphous phase, while the α process is attributed to the movement of chains in the crystalline phase. Regarding DMA results for HDPE and PP, this mechanical relaxation is observed around 350 K. For both polymers, a decrease in elastic components with temperature is observed, up to the flow temperature, representing a nonlinear dependency of the elastic modulus. A maximum in tan δ is observed involving maximum energy dissipation (viscous contribution) when the flow process begins.

### 4.2. Stress Relaxation

**Figure 6:**
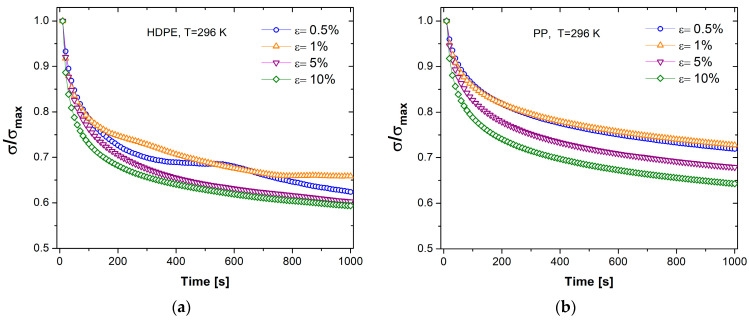
Relative stress $\sigma /\sigma_{max}$ as a function of time for (**a**) HDPE and (**b**) PP.

The stress relaxation experiment consists of applying a constant step strain for a certain time under isothermal conditions. Figure 6a,b shows the results for stress relaxation tests in HDPE and PP, normalized concerning the maximum value. A tendency for relative stress to decrease is observed as strain increases. The behavior reflected at low strains is more complex for HDPE compared to PP, which is related to its semicrystalline nature [65,66]. Under the same strain and time conditions of ε = 10% and t = 1000 s for HDPE, the initial stress decreases by 40.7%, while for PP, there is a decrease of 35.7%. This means that there is greater energy dissipation in the HDPE relaxation.

Also, in each case, the stress decreases as time passes. This behavior is because polymer chains tend to increase their entropy, which was reduced when the material was abruptly deformed. The decrease in stress over time is a macroscopic manifestation of the transition from stretched or aligned polymer chains, with lower entropy, to a random coil conformation, where entropy is maximized.

### 4.3. Mathematical Modeling

**Figure 7:**
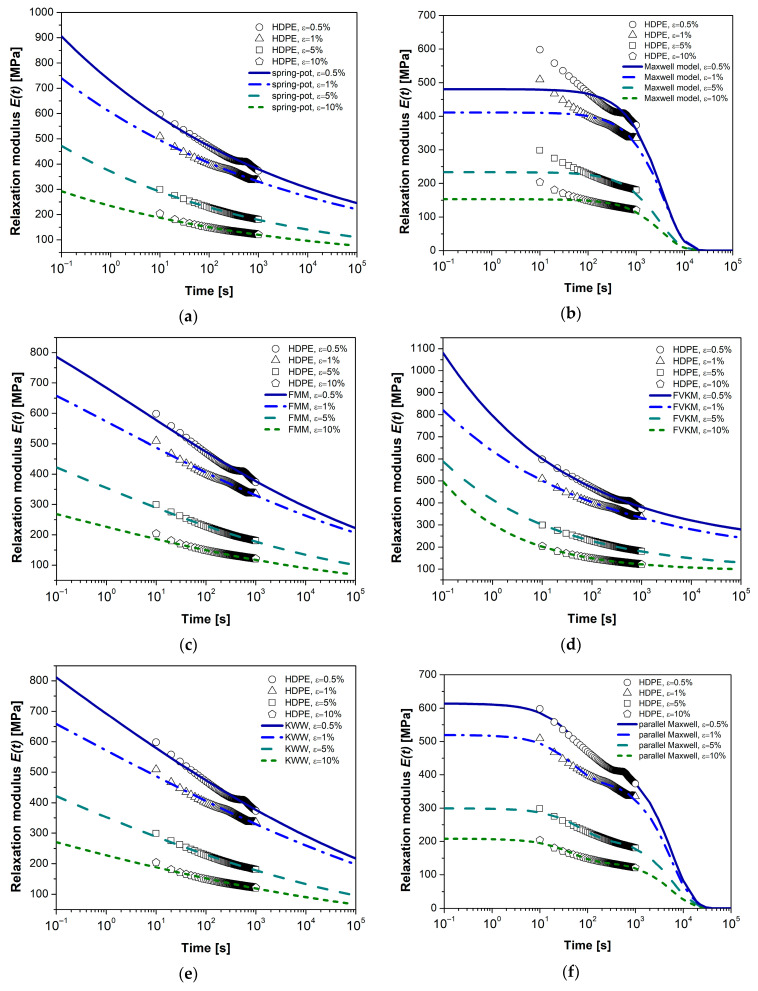
Mathematical modeling of relaxation modulus plots, comparison of HDPE for (**a**) spring-pot model, (**b**) classical Maxwell model, (**c**) Fractional Maxwell model, (**d**) Fractional Voigt–Kelvin model, (**e**) KWW model, and (**f**) parallel Maxwell model.

**Figure 8:**
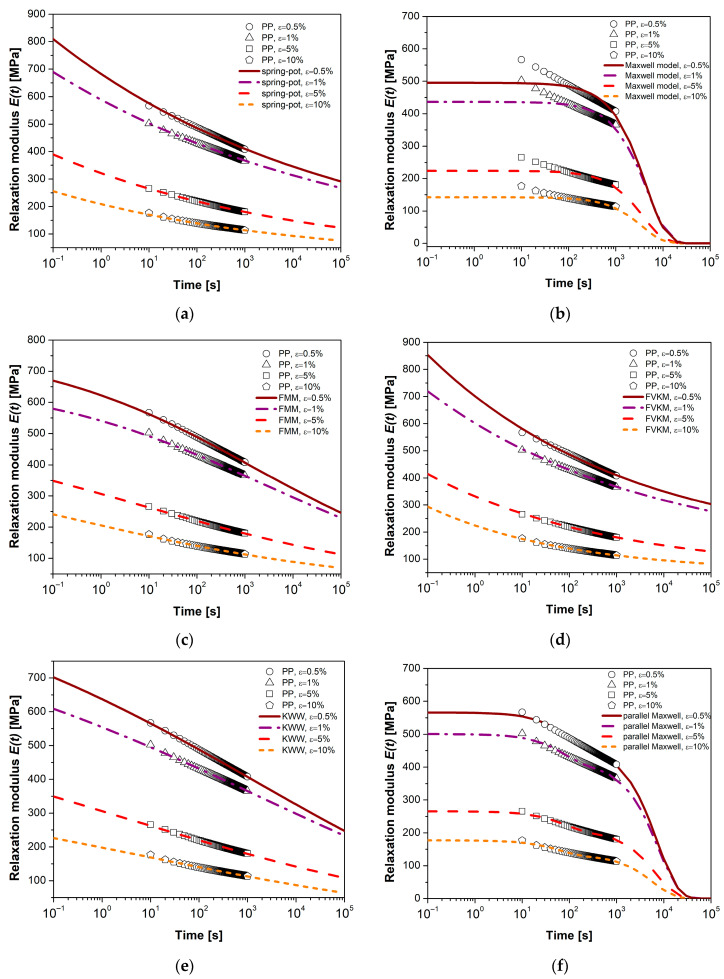
Mathematical modeling of relaxation modulus plots, comparison of PP for (**a**) spring-pot model, (**b**) classical Maxwell model, (**c**) Fractional Maxwell model, (**d**) Fractional Voigt–Kelvin model, (**e**) KWW model, and (**f**) parallel Maxwell model.

The models used in this study are based on three definitions of derivatives (classical, fractional, and conformable) and consist of two parameters (Maxwell), three parameters (SP, FMM, FVKM, and KWW), and four parameters (parallel Maxwell, PM, with two Maxwell elements). Some authors have proposed this last classical model to address the stress relaxation of semicrystalline polymers [65]. The idea is that each Maxwell element is destined for the amorphous and crystalline phases, respectively. Figure 7 and Figure 8 show the experimental relaxation curves for HDPE and PP. Each figure individually displays the response of each viscoelastic model, facilitating visual comparison of the results. Table 1, Table 2, Table 3, Table 4, Table 5 and Table 6 detail the parameters of the viscoelastic models (elastic modulus *E*, relaxation time τ, and fractional order α) along with the statistical descriptors *R*^2^, *AAD*, and *MSE*.

In the literature, it is common to use the correlation coefficient *R*^2^ to determine the most suitable model for representing the viscoelastic behavior of materials [29]. The models evaluated in this study exhibit *R*^2^ values ranging from 0.97 to 0.99, except for the classical Maxwell model, whose values range between 0.67 and 0.82. For practical purposes, an *R*^2^ value greater than 0.9 is recommended, which suggests a good correspondence between experimental data and model predictions. Based on this criterion, the classical Maxwell model is the least accurate in describing stress relaxation tests due to its integer-order differential operator and the exponential function used as its analytical solution. However, adding a finite number of Maxwell elements in parallel can improve the representation of viscoelastic behavior, although this introduces parameters that lack clear physical interpretation.

Following the *R*^2^ criterion, the FVKM and SP models provide the best fit to the experimental data for HDPE, while for PP, the best-performing models are SP and FMM. When considering *AAD* as the statistical criterion, aiming for a value as close to zero as possible, the representative models for HDPE are FVKM and SP, while for PP, they are SP and KWW. Regarding *MSE*, the ideal models for HDPE are FVKM and PM, while for PP, they are KWW and FMM. Based on these results, it can be concluded that the SP, FMM, FVKM, and KWW models (with three parameters) are suitable for representing stress relaxation in polymeric materials. Specifically, the FMM model presents a solution involving the Mittag–Leffler function, which generalizes the exponential function when α = 1. This function exhibits two interesting asymptotic behaviors: at short times, it behaves like a stretched exponential function (KWW function); at long times, it behaves like a power law (FVKM solution) [49,67,68]. These characteristics explain the similarities observed among these models.

The results of the parameters *E* and τ for the proposed models in the analysis show no direct relationship between these parameters and the applied deformation level. However, a trend is observed where *E* and τ decrease as the deformation level increases, suggesting a greater manifestation of the viscous component.

**Figure 9:**
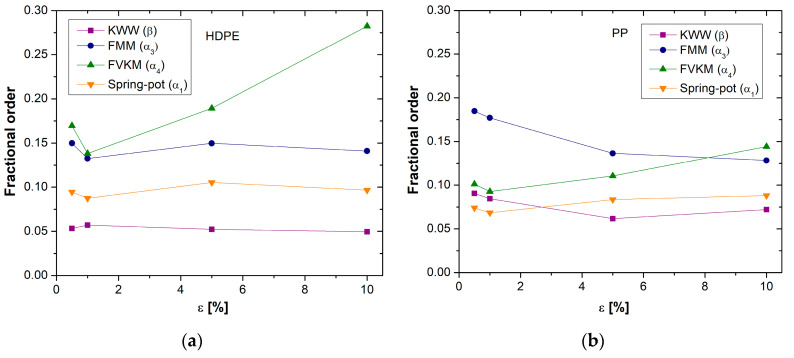
Fractional order comparison in models as a function of strain for (**a**) HDPE and (**b**) PP.

For the fractional order, the classical Maxwell and Voigt–Kelvin models present a value equal to 1. In contrast, for the SP, FMM, FVKM, and KWW models, the fractional order only takes values between 0 and 1. Figure 9 shows a graph of the fractional order as a function of the deformation level for HDPE and PP, respectively. It is observed that the values are closer to 0, indicating the characteristic behavior of a viscoelastic solid. However, there is not a notable relationship between the two parameters. In the case of the FMM, the fractional order tends to increase with higher deformation levels, reflecting increased energy dissipation. Conversely, for FVKM, the fractional order decreases as the deformation level increases, indicating lower energy dissipation. The β value considered in the KWW model is the lowest for both polymers.

The literature does not provide a clear explanation for the differences in fractional order values obtained. It is presumed that this could be due to the arrangement of the constitutive elements in the models (series vs. parallel) and the type of derivative definition used. This suggests that energy storage and dissipation manifest at different time scales, leading to differences in fractional order values.

### 4.4. Statistical Analysis

The design and optimization of these results are related to analyzing the goodness of fit metrics (*R*^2^, *AAD*, and *MSE*) for each model and validating the application of MANOVA and RSM in the polymer relaxation plots. From the above, it will be possible to analyze the impact of two main factors, specifically mathematical models and strain, on the three response variables, as shown in Table 7. This approach includes both categorical factors and continuous variables to establish its effectiveness, accuracy, and applicability in the selection of adequate models and how to optimize the response [69].

The response variables *R*^2^, *AAD*, and *MSE* are considered representative of the fit quality in each of the mathematical models evaluated for the two polymers (HDPE and PP) separately. Given that the responses are significantly correlated, as confirmed by the correlation analysis, MANOVA enables us to determine whether the use of different mathematical models, along with varying levels of strain, significantly influences the fit quality of the measurements, optimizing the responses when no significant curvatures are observed in the analysis of variance (ANOVA) [70].

Table 8 shows the results of MANOVA for HDPE where the Wilks’ lambda statistic was 0.35309, indicating a significant multivariate effect of strain on the dependent variables, with an approximate F-value of 1.885 and a *p*-value of 0.092. Although the *p*-value suggests a trend towards significance, it does not reach the conventional threshold of 0.05, indicating that the effect of “strain” may not be statistically significant. The Lawley–Hotelling statistics yielded a value of 1.39451 (F = 1.808, *p* = 0.102), and Pillai’s trace was 0.80169 (F = 1.823, *p* = 0.090), further suggesting that while there is some evidence of an effect, it does not provide conclusive support for the significance of “strain”. In contrast, the MANOVA results for the Model factor showed more compelling evidence of significance. The Wilks’ lambda statistic was extremely low at 0.00303, corresponding to a highly significant approximate F-value of 17.353 (*p* < 0.0001). The Lawley–Hotelling trace statistic was 182.68714 (F = 142.090, *p* < 0.0001), indicating a robust effect of the “model” factor on the dependent variables. Similarly, Pillai’s trace (1.44117, F = 2.774, *p* = 0.004) and Roy’s largest root (181.88317) confirm that the “model” significantly influences the response variables.

In the case of PP, Table 9 shows the MANOVA results for strain where the Wilks’ lambda statistic was 0.43090, yielding an approximate F-value of 1.460 (*p* = 0.207). This suggests that there is no statistically significant multivariate effect of strain on the dependent variables, as the *p*-value exceeds the conventional threshold of 0.05. Additionally, the Lawley–Hotelling trace statistic was 1.04104 (F = 1.350, *p* = 0.248), and Pillai’s trace was 0.69341 (F = 1.503, *p* = 0.176), further supporting the conclusion that the “strain” factor does not significantly influence the responses in this context. Roy’s largest root statistic of 0.63802 reinforces the lack of significant effects observed for “strain”.

Conversely, the MANOVA results for the “model” factor indicated a significant multivariate effect. The Wilks’ lambda statistic was extremely low at 0.00054, resulting in an approximate F-value of 34.511 (*p* < 0.0001). This strong evidence of significance suggests that the model factor has a substantial impact on the dependent variables. The Lawley–Hotelling trace statistic was 1138.97153 (F = 885.867, *p* < 0.0001), confirming the robustness of the Model factor’s effect. Furthermore, Pillai’s trace statistic was 1.38408 (F = 2.570, *p* = 0.007), indicating significant contributions of the “model” to the response variables. Roy’s largest root statistic of 1138.34612 further substantiates the strong influence of the “model” factor.

**Figure 10:**
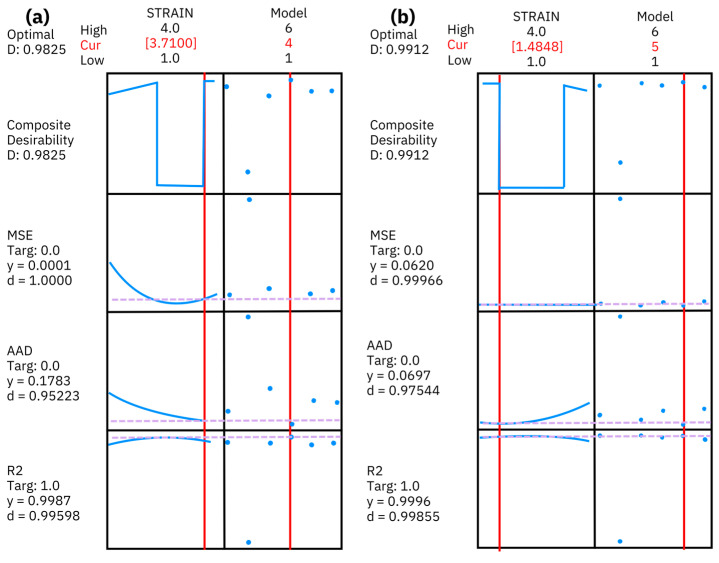
Optimization plot from RSM: (**a**) HDPE and (**b**) PP.

From the MANOVA results for both polymers, HDPE and PP, the strain does not show a statistically significant effect on the response variables, while the model factor exhibits a highly significant impact, suggesting that further investigation into its specific contributions is warranted. In this case, the response surface methodology (RSM) was employed to further explore the interaction effects between the factors on the response variables, facilitate the optimization of complex processes by fitting a suitable model to the data, and provide insights into the relationships among variables [71]. By analyzing the response surfaces, we were able to visualize the effects of varying strain levels and mathematical models on the quality measures. This approach is particularly useful for identifying the optimal conditions and achieving the desired outcomes in polymer analysis [72]. The analysis not only reveals potential peaks in response variables but also helps in understanding the curvature of the response surfaces, which can guide decision making in polymer selection and processing. Overall, RSM complements the insights gained from MANOVA, allowing for a comprehensive evaluation of the factors influencing the performance of the two polymers, as shown in Figure 10.

In Figure 10a, the RSM reveals the optimization plot where the composite desirability reached a score of 0.9825, indicating that the minimum MSE was achieved with a value of 0.00001, the *AAD* was minimized to 0.1783, and the R^2^ was maximized at 0.9987. These results suggest that the mathematical model significantly influences the responses, guiding us to the Fractional Voigt–Kelvin as the most effective model for characterizing HDPE. In the analysis of the PP sample (Figure 10b), the R^2^ value was recorded at 0.9996, the *AAD* was minimized to 0.0697, and the MSE was calculated at 0.0620.

These results suggest that the adjustments made during the optimization process have led to the KWW mathematical model, providing reliable insights into the phenomena being studied. In both optimization plots, the desirability score is above 0.95, which suggests that the mathematical model significantly impacts the responses, steering us toward an effective characterization of the HDPE and PP using these conditions.

Fractional viscoelastic models have proven to be highly effective in predicting the viscoelastic behavior of polymeric materials with fewer parameters compared to conventional models. For instance, previous studies have demonstrated their ability to accurately describe the mechanical response of complex materials, reinforcing their relevance in advanced material characterization [29]. The main challenge in applying both integer-order and fractional-order viscoelastic models lies in accurately determining their parameters. In this regard, the response surface methodology (RSM) has emerged as a powerful and efficient approach for predicting rheological and mechanical characteristics across various material systems. This technique has been successfully applied to optimize the relationship between fractional model parameters in asphalt-based materials, demonstrating its robustness in improving viscoelastic modeling. Moreover, its effectiveness extends beyond asphalt systems, as evidenced by its application in hydrogel formulations, where the optimization of colloidal particle interactions significantly enhanced their viscoelastic properties [73]. These findings underscore the potential of integrating fractional calculus with statistical optimization techniques to enhance the predictive capabilities of viscoelastic models in diverse polymeric systems. The high desirability scores obtained in this study further validate the effectiveness of these methodologies, reinforcing their applicability in the precise characterization of HDPE and PP under varying conditions.

## 5. Conclusions

In this study, we evaluated the viscoelastic response of HDPE and PP through stress relaxation tests at different strain levels. The primary objective was to compare three derivative definitions—classical, fractional, and conformable—and employ statistical analysis to identify the most accurate viscoelastic models for these polymers. Our findings confirm that fractional and conformable derivative models provide a more accurate representation of the viscoelastic behavior of HDPE and PP compared to classical models.

The semi-crystalline structure of the studied polymers was confirmed through FTIR, TGA, DSC, and DMA analyses, providing a solid foundation for understanding their molecular behavior. Stress relaxation tests revealed that the viscous component becomes more significant at higher strain levels, as evidenced by the decrease in elastic modulus and relaxation time.

Among the models tested, the classical Maxwell model showed the lowest accuracy, highlighting the limitations of traditional viscoelastic models in capturing the complex behaviors of HDPE and PP. In contrast, fractional models performed better, with fractional order values approaching 0, which indicated a viscoelastic solid-like behavior for both polymers. Notably, the Fractional Maxwell (FMM) and Fractional Voigt–Kelvin (FVKM) models demonstrated significant variation in fractional order with strain, underlining their potential for accurately describing energy dissipation and molecular mobility.

The spring-pot, FMM, FVKM, and Kohlrausch–Williams–Watts (KWW) models provided the best fit to the experimental data according to statistical criteria. The FVKM was found to be the most suitable for HDPE, while the KWW model performed best for PP. Furthermore, the use of the response surface methodology (RSM) and multivariate analysis of variance (MANOVA) validated the importance of selecting the appropriate mathematical models for accurately representing the viscoelastic properties of these materials.

These results underscore the potential of fractional and conformable derivative models to improve the understanding and prediction of viscoelastic behavior in polymers. By fulfilling the study’s objective of identifying the most effective models for HDPE and PP, our findings have notable implications for material design—particularly in industries such as packaging, automotive, and medical devices, where the long-term mechanical performance of polymers is critical. In future research, it will be important to extend these methods to other polymer systems and further explore the physical interpretation of fractional parameters, which could enhance their applicability in material design and structural analysis.

## Figures and Tables

**Table 1:** Modeling parameters for HDPE and PP with spring-pot model.

$\mathrm{Spring}\text{-}\mathrm{Pot}\;\text{→}\;E\left(t\right)=\left[\frac{E_{1}}{\Gamma \left(1-\alpha_{1}\right)}\right]{\left(\frac{t}{\tau_{1}}\right)}^{-\alpha_{1}}$							
	ε (%)	$E_{1}$ (MPa)	$\tau_{1}$ (s)	$\alpha_{1}$	*R* ^2^	*AAD*	*MSE*
HDPE	0.5	966.7	0.0964	0.0944	0.9821	1.0938	28.094
	1	796.5	0.0822	0.0874	0.9912	0.6301	8.3520
	5	533.8	0.0605	0.1052	0.9953	0.5221	2.0940
	10	424.7	0.0040	0.0965	0.9791	0.6032	3.6810
PP	0.5	464.5	346.80	0.0739	0.9972	0.2682	2.7908
	1	604.0	1.3120	0.0684	0.9987	0.2117	0.9027
	5	292.7	5.8560	0.0834	0.9996	0.1249	0.1232
	10	274.7	0.0860	0.0881	0.9960	0.1192	0.4106

**Table 2:** Modeling parameters for HDPE and PP with classical Maxwell model.

$\mathrm{Classical}\;\mathrm{Maxwell}\;\text{→}\;{E\left(t\right)=E}_{2}\exp\;\left[-\left(\frac{t}{\tau_{2}}\right)\right]$						
	ε (%)	$E_{2}$ (MPa)	$\tau_{2}$ (s)	*R* ^2^	*AAD*	*MSE*
HDPE	0.5	480.2	3467.4	0.7354	2.9496	420.449
	1	411.3	3785.0	0.7541	2.7007	236.404
	5	233.4	3101.7	0.7163	3.7321	128.638
	10	153.0	3401.4	0.6708	3.4611	58.6790
PP	0.5	495.0	4323.3	0.8214	2.1191	184.483
	1	436.6	4732.6	0.8102	1.9392	130.282
	5	223.3	3844.7	0.7819	2.6075	58.1607
	10	142.3	3636.4	0.7520	2.8389	30.6643

**Table 3:** Modeling parameters for HDPE and PP with Fractional Maxwell model.

	$\mathrm{Fractional}\;\mathrm{Maxwell}\;\text{→}\;{E\left(t\right)=E}_{3}M_{\alpha }\left[-{\left(\frac{t}{\tau_{3}}\right)}^{\alpha_{3}}\right]$						
	ε (%)	$E_{3}($MPa)	$\tau_{3}$ (s)	$\alpha_{3}$	*R* ^2^	*AAD*	*MSE*
HDPE	0.5	1225.7	8.2690	0.1499	0.9766	1.1838	37.079
	1	1116.6	2.6149	0.1325	0.9878	1.2579	29.173
	5	775.52	0.5711	0.1498	0.9916	0.8218	3.9980
	10	511.97	0.3456	0.141	0.9747	1.5241	8.0540
PP	0.5	779.3	2838.6	0.1848	0.9993	0.1373	0.7072
	1	674.2	4435.7	0.1771	0.9974	0.1697	1.7351
	5	553.1	8.6310	0.1365	0.9990	0.1940	0.2543
	10	483.8	0.1694	0.1283	0.9956	0.5300	0.7575

**Table 4:** Modeling parameters for HDPE and PP with Fractional Voigt–Kelvin model.

$\mathrm{Fractional}\;\mathrm{Voigt}\text{-}\mathrm{Kelvin}\;\to \;E\left(t\right)=E_{4}\left[1+\frac{{\left(\frac{t}{\tau_{4}}\right)}^{-\alpha_{4}}}{\Gamma \left(1-\alpha_{4}\right)}\right]$							
	ε (%)	$E_{4}$ (MPa)	$\tau_{4}$ (s)	$\alpha_{4}$	*R* ^2^	*AAD*	*MSE*
HDPE	0.5	195.0	1558.0	0.1697	0.985	1.0245	23.824
	1	141.7	16,860	0.1382	0.9924	0.6318	7.2060
	5	93.38	1425.0	0.1894	0.9995	0.1981	0.2220
	10	91.38	45.520	0.2825	0.9991	0.1977	0.1550
PP	0.5	122.5	8.950 × 10^6^	0.1012	0.9950	0.3622	5.1947
	1	106.4	3.149 × 10^7^	0.09257	0.9974	0.2808	1.7368
	5	48.41	1.748 × 10^7^	0.1105	0.9994	0.1380	0.1691
	10	48.96	1.420 × 10^4^	0.1442	0.9994	0.1324	0.0791

**Table 5:** Modeling parameters for HDPE and PP with KWW model.

$\mathrm{KWW}\;\text{→}\;E\left(t\right)=E_{5}\exp\;\left[-\frac{1}{\beta }{\left(\frac{t}{\tau_{5}}\right)}^{\beta }\right]$							
	ε (%)	$E_{5}$ (M Pa)	$\tau_{5}$ (s)	β	*R* ^2^	*AAD*	*MSE*
HDPE	0.5	2716	2 × 10^21^	0.0533	0.9772	1.1788	36.285
	1	1792	6 × 10^20^	0.0570	0.9869	0.6669	12.499
	5	1699	5 × 10^20^	0.0523	0.9901	0.8504	4.4620
	10	1121	2 × 10^22^	0.0494	0.9710	1.0580	4.9670
PP	0.5	1066	5 × 10^14^	0.0905	0.9990	0.0661	0.1411
	1	938.2	1 × 10^16^	0.0845	0.9989	0.1037	0.7714
	5	836.1	4 × 10^19^	0.0616	0.9986	0.2326	0.3608
	10	474.9	5 × 10^16^	0.0719	0.9917	0.4917	1.0143

**Table 6:** Modeling parameters for HDPE and PP with parallel Maxwell model.

	$\mathrm{Parallel}\;\mathrm{Maxwell}\;\text{→}\;E\left(t\right)=E_{6}\exp\;\left[-\left(\frac{t}{\tau_{6}}\right)\right]+E_{7}\exp\;\left[-\left(\frac{t}{\tau_{7}}\right)\right]$							
	ε (%)	$E_{6}$ (MPa)	$\tau_{6}$ (s)	$E_{7}$ (MPa)	$\tau_{7}$ (s)	*R* ^2^	*AAD*	*MSE*
HDPE	0.5	170.4	57.800	443.5	5941.77	0.9925	0.6702	11.927
	1	132.2	48.008	387.5	5733.90	0.9778	1.1073	20.888
	5	89.71	69.930	209.8	6119.95	0.9900	0.7341	4.4390
	10	67.27	48.100	140.8	5945.30	0.9836	0.9102	2.8690
PP	0.5	102.8	86.206	463.2	7429.42	0.9951	0.3699	5.0922
	1	87.78	74.571	412.9	7710.10	0.9922	0.4117	5.2450
	5	58.88	76.982	206.7	6821.30	0.9930	0.4929	1.8210
	10	44.87	61.050	132.0	6116.20	0.9879	0.6854	1.4702

**Table 7:** Data set for the design of experiment.

Factor	Natural Level	Code Level	Response Variables	Pearson Correlation		
Strain	0.5%	1	*R^2^*, *AAD*, *MSE*	HDPE		
	1%	2			*R^2^*	*AAD*
	5%	3		*AAD*	−0.955	---
	10%	4		*MSE*	−0.736	0.692
Model	Spring-pot	1				
	Classical Maxwell	2		PP		
	Fractional Maxwell	3			*R^2^*	*AAD*
	Fractional Voigt-Kelvin	4		*AAD*	−0.985	---
	KWW	5		*MSE*	−0.748	0.717
	Parallel Maxwell	6				

**Table 8:** MANOVA for HDPE.

**MANOVA for Strain**					
**Criterion**	**Test Stat**	**Approx F**	**DF**		***p***
			**Num**	**Den**	
Wilks’	0.35309	1.885	9	31	0.092
Lawley-Hotelling	1.39451	1.808	9	35	0.102
Pillai’s	0.80169	1.823	9	45	0.090
Roy’s	0.92097	-	-	-	-
**MANOVA for Model**					
**Criterion**	**Test Stat**	**Approx F**	**DF**		***p***
			**Num**	**Den**	
Wilks’	0.00303	17.353	15	36	0.000
Lawley-Hotelling	182.68714	142.090	15	35	0.000
Pillai’s	1.44117	2.774	15	45	0.004
Roy’s	181.88317	-	-	-	-

**Table 9:** MANOVA for PP.

**MANOVA for Strain**					
**Criterion**	**Test Stat**	**Approx F**	**DF**		***p***
			**Num**	**Den**	
Wilks’	0.43090	1.460	9	31	0.207
Lawley-Hotelling	1.04104	1.350	9	35	0.248
Pillai’s	0.69341	1.503	9	45	0.176
Roy’s	0.63802				
**MANOVA for Model**					
**Criterion**	**Test Stat**	**Approx F**	**DF**		***p***
			**Num**	**Den**	
Wilks’	0.00054	34.511	15	36	0.000
Lawley-Hotelling	1138.97153	885.867	15	35	0.000
Pillai’s	1.38408	2.570	15	45	0.007
Roy’s	1138.34612				

## Data Availability

The original contributions presented in the study are included in the article, and further inquiries can be directed to the corresponding author.

## References

[1] Zanchin G., Leone G. (2021). Polyolefin Thermoplastic Elastomers from Polymerization Catalysis: Advantages, Pitfalls and Future Challenges. Prog. Polym. Sci..

[2] Gebrehiwot S.Z., Espinosa-Leal L. (2022). Characterising the Linear Viscoelastic Behaviour of an Injection Moulding Grade Polypropylene Polymer. Mech. Time-Depend. Mater..

[3] Rentería-Baltiérrez F.Y., Reyes-Melo M.E., López-Walle B., García-Loera A.F., González-González V.A. (2020). A Fractional Calculus Approach to Study Mechanical Relaxations on Hybrid Films of Fe2O3 Nanoparticles and Polyvinyl Butyral. J. Therm. Anal. Calorim..

[4] Rentería-Baltiérrez F.Y., Reyes-Melo M.E., Puente-Córdova J.G., López-Walle B. (2021). Correlation between the Mechanical and Dielectric Responses in Polymer Films by a Fractional Calculus Approach. J. Appl. Polym. Sci..

[5] Pizzanelli S., Prevosto D., Labardi M., Guazzini T., Bronco S., Forte C., Calucci L. (2017). Dynamics of Poly(Vinyl Butyral) Studied Using Dielectric Spectroscopy and 1 H NMR Relaxometry. Phys. Chem. Chem. Phys..

[6] Calucci L., Pizzanelli S., Mandoli A., Birczyński A., Lalowicz Z.T., De Monte C., Ricci L., Bronco S. (2021). Unravelling Main- and Side-Chain Motions in Polymers with NMR Spectroscopy and Relaxometry: The Case of Polyvinyl Butyral. Polymers.

[7] Ornaghi H.L., Almeida J.H.S., Monticeli F.M., Neves R.M. (2020). Stress Relaxation, Creep, and Recovery of Carbon Fiber Non-Crimp Fabric Composites. Compos. Part C Open Access.

[8] Neves R.M., Ornaghi H.L., Alves F.C., Zattera A.J., Tom M., Lal H.M., Uthaman A., Thomas S. (2023). Creep and Stress Relaxation Behavior of Functionalized Microcrystalline Cellulose/Epoxy Composites. Cellulose.

[9] Nam T.H., Petríková I., Marvalová B. (2021). Experimental and Numerical Research of Stress Relaxation Behavior of Magnetorheological Elastomer. Polym. Test..

[10] Bonfanti A., Kaplan J.L., Charras G., Kabla A. (2020). Fractional Viscoelastic Models for Power-Law Materials. Soft Matter.

[11] Stankiewicz A. (2023). On Applicability of the Relaxation Spectrum of Fractional Maxwell Model to Description of Unimodal Relaxation Spectra of Polymers. Polymers.

[12] Schmidt R.F., Winter H.H., Gradzielski M. (2024). Generalized vs. Fractional: A Comparative Analysis of Maxwell Models Applied to Entangled Polymer Solutions. Soft Matter.

[13] Jóźwiak B., Orczykowska M., Dziubiński M. (2015). Fractional Generalizations of Maxwell and Kelvin-Voigt Models for Biopolymer Characterization. PLoS ONE.

[14] Meng R. (2021). Application of Fractional Calculus to Modeling the Nonlinear Behaviors of Ferroelectric Polymer Composites: Viscoelasticity and Dielectricity. Membranes.

[15] Reyes-Melo M.E., Martínez-Vega J.J., Guerrero-Salazar C.A., Ortiz-Méndez U. (2006). Mechanical and Dielectric Relaxation Phenomena of Poly(Ethylene-2,6- Napthalene Dicarboxylate) by Fractional Calculus Approach. J. Appl. Polym. Sci..

[16] Atangana A., Gómez-Aguilar J.F. (2018). Decolonisation of Fractional Calculus Rules: Breaking Commutativity and Associativity to Capture More Natural Phenomena. Eur. Phys. J. Plus.

[17] Abro K.A., Memon A.A., Uqaili M.A. (2018). A Comparative Mathematical Analysis of RL and RC Electrical Circuits via Atangana-Baleanu and Caputo-Fabrizio Fractional Derivatives. Eur. Phys. J. Plus.

[18] Sun H., Zhang Y., Baleanu D., Chen W., Chen Y. (2018). A New Collection of Real World Applications of Fractional Calculus in Science and Engineering. Commun. Nonlinear Sci. Numer. Simul..

[19] Podlubny I. (2002). Geometric and Physical Interpretation of Fractional Integration and Fractional Differentiation. Fract. Calc. Appl. Anal..

[20] Moshrefi-Torbati M., Hammond J.K. (1998). Physical and Geometrical Interpretation of Fractional Operators. J. Frankl. Inst..

[21] Du M., Wang Z., Hu H. (2013). Measuring Memory with the Order of Fractional Derivative. Sci. Rep..

[22] Ruby, Mandal M. (2024). The Geometrical and Physical Interpretation of Fractional Order Derivatives for a General Class of Functions. Math. Methods Appl. Sci..

[23] Rentería-Baltiérrez F.Y., Reyes-Melo M.E., Puente-Córdova J.G., López-Walle B. (2023). Application of Fractional Calculus in the Mechanical and Dielectric Correlation Model of Hybrid Polymer Films with Different Average Molecular Weight Matrices. Polym. Bull..

[24] Reyes-Melo E., Martinez-Vega J., Guerrero-Salazar C., Ortiz-Mendez U. (2005). Application of Fractional Calculus to the Modeling of Dielectric Relaxation Phenomena in Polymeric Materials. J. Appl. Polym. Sci..

[25] Reyes-Melo M.E., Garza-Navarro M.A., González-González V.A., Guerrero-Salazar C.A., Martínez-Vega J., Ortiz-Méndez U. (2009). Application of Fractional Calculus to the Modeling of the Complex Magnetic Susceptibility for Polymeric-magnetic Nanocomposites Dispersed into a Liquid Media. J. Appl. Polym. Sci..

[26] Mammeri S., Bouacha K., Chaoui K., Ghabeche W., Berkas K. (2024). Filament Manufacturing via External Grooving of an HDPE Pipe Wall: RSM Optimization and Mechanical Tests Validation. Res. Eng. Struct. Mater..

[27] Jones D.S., Yu T., Andrews G.P. (2019). A Statistical Determination of the Contribution of Viscoelasticity of Aqueous Carbohydrate Polymer Networks to Drug Release. Carbohydr. Polym..

[28] Toapanta O.G., Paredes J., Meneses M., Salinas G. (2024). Validation of DOE Factorial/Taguchi/Surface Response Models of Mechanical Properties of Synthetic and Natural Fiber Reinforced Epoxy Matrix Hybrid Material. Polymers.

[29] Hajikarimi P., Ehsani M., EL Haloui Y., Fakhari Tehrani F., Absi J., Moghadas Nejad F. (2022). Fractional Viscoelastic Modeling of Modified Asphalt Mastics Using Response Surface Method. Constr. Build. Mater..

[30] Ovalle-Flores L., Rodríguez-Nieto M., Zárate-Triviño D., Rodríguez-Padilla C., Menchaca J.L. (2023). Methodologies and Models for Measuring Viscoelastic Properties of Cancer Cells: Towards a Universal Classification. J. Mech. Behav. Biomed. Mater..

[31] Braun J., Bernarding J., Snellings J., Meyer T., Dantas de Moraes P.A., Safraou Y., Wells R.G., Guo J., Tzschätzsch H., Zappe A. (2024). On the Relationship between Viscoelasticity and Water Diffusion in Soft Biological Tissues. Acta Biomater..

[32] Puente-Córdova J.G., Reyes-Melo M.E., Palacios-Pineda L.M., Martínez-Perales I.A., Martínez-Romero O., Elías-Zúñiga A. (2018). Fabrication and Characterization of Isotropic and Anisotropic Magnetorheological Elastomers, Based on Silicone Rubber and Carbonyl Iron Microparticles. Polymers.

[33] Puente-Córdova J.G., Segura-Méndez K.L. (2024). Comparative Study of the Viscoelastic Behavior on PLA Filaments, a Fractional Calculus Approach. Complex Systems and Their Applications.

[34] Roland C.M. (2010). Relaxation Phenomena in Vitrifying Polymers and Molecular Liquids. Macromolecules.

[35] Etienne S., Hazeg N., Duval E., Mermet A., Wypych A., David L. (2007). Physical Aging and Molecular Mobility of Amorphous Polymers. J. Non. Cryst. Solids.

[36] Carini G., Bartolotta A., Carini G., D’Angelo G., Federico M., Di Marco G. (2018). Water-Driven Segmental Cooperativity in Polyvinyl Butyral. Eur. Polym. J..

[37] Reyes-Melo E., Martinez-Vega J., Guerrero-Salazar C., Ortiz-Mendez U. (2004). On the modeling of the dynamic-elastic modulus for polymer materials under isochronal conditions. J. Appl. Polym. Sci..

[38] Meneses A., Naya S., Francisco-Fernández M., López-Beceiro J., Gracia-Fernández C., Tarrío-Saavedra J. (2023). TTS Package: Computational Tools for the Application of the Time Temperature Superposition Principle. Heliyon.

[39] Medina-Torres L., Nuñez-Ramirez D.M., Cabrales-Gonzalez A.M., Manero O. (2024). Master Curves Obtained by Time–Temperature–Concentration Double Superposition of the Κ-carrageenan Gelling Biopolymer. J. Food Process Eng..

[40] Genovese A., Farroni F., Sakhnevych A. (2022). Fractional Calculus Approach to Reproduce Material Viscoelastic Behavior, Including the Time–Temperature Superposition Phenomenon. Polymers.

[41] Koeller R.C. (1984). Applications of Fractional Calculus to the Theory of Viscoelasticity. J. Appl. Mech..

[42] Miranda-Valdez I.Y., Sourroubille M., Mäkinen T., Puente-Córdova J.G., Puisto A., Koivisto J., Alava M.J. (2024). Fractional Rheology of Colloidal Hydrogels with Cellulose Nanofibers. Cellulose.

[43] Miranda-Valdez I.Y., Puente-Córdova J.G., Rentería-Baltiérrez F.Y., Fliri L., Hummel M., Puisto A., Koivisto J., Alava M.J. (2024). Viscoelastic Phenomena in Methylcellulose Aqueous Systems: Application of Fractional Calculus. Food Hydrocoll..

[44] Heymans N., Bauwens J.-C. (1994). Fractal Rheological Models and Fractional Differential Equations for Viscoelastic Behavior. Rheol. Acta.

[45] Heymans N. (1996). Hierarchical Models for Viscoelasticity: Dynamic Behaviour in the Linear Range. Rheol. Acta.

[46] Faber T.J., Jaishankar A., McKinley G.H. (2017). Describing the Firmness, Springiness and Rubberiness of Food Gels Using Fractional Calculus. Part I: Theoretical Framework. Food Hydrocoll..

[47] Song J., Holten-Andersen N., McKinley G.H. (2023). Non-Maxwellian Viscoelastic Stress Relaxations in Soft Matter. Soft Matter.

[48] Alcoutlabi M., Martinez-Vega J.J. (1998). Application of Fractional Calculus to Viscoelastic Behaviour Modelling and to the Physical Ageing Phenomenon in Glassy Amorphous Polymers. Polymers.

[49] Mainardi F., Spada G. (2011). Creep, Relaxation and Viscosity Properties for Basic Fractional Models in Rheology. Eur. Phys. J. Spec. Top..

[50] Khalil R., Al Horani M., Yousef A., Sababheh M. (2014). A New Definition of Fractional Derivative. J. Comput. Appl. Math..

[51] Puente-Córdova J.G., Rentería-Baltiérrez F.Y., Reyes-Melo M.E. (2020). La Derivada Conformable y Sus Aplicaciones En Ingeniería. Ingenierias.

[52] Kachhia K.B., Gosai D.A. (2024). Conformable Derivative Models for Linear Viscoelastic Materials. Mech. Time-Depend. Mater..

[53] Verdurmen-Noël L., Baldo L., Bremmers S. (2001). SEC–FTIR Characterization of Semi-Crystalline HDPE and PP. Polymers.

[54] Gulmine J., Janissek P., Heise H., Akcelrud L. (2002). Polyethylene Characterization by FTIR. Polym. Test..

[55] Gopanna A., Mandapati R.N., Thomas S.P., Rajan K., Chavali M. (2019). Fourier Transform Infrared Spectroscopy (FTIR), Raman Spectroscopy and Wide-Angle X-Ray Scattering (WAXS) of Polypropylene (PP)/Cyclic Olefin Copolymer (COC) Blends for Qualitative and Quantitative Analysis. Polym. Bull..

[56] Ichim M., Stelea L., Filip I., Lisa G., Muresan E.I. (2022). Thermal and Mechanical Characterization of Coir Fibre–Reinforced Polypropylene Biocomposites. Crystals.

[57] Ravi M., Song S.-H., Gu K.-M., Tang J.-N., Zhang Z.-Y. (2015). Effect of Lithium Thiocyanate Addition on the Structural and Electrical Properties of Biodegradable Poly(ε-Caprolactone) Polymer Films. Ionics.

[58] Al-Bayaty S.A., Al-Uqaily R.A.H., Hameed S. (2020). Study of Thermal Degradation Kinetics of High Density Polyethlyene (HDPE) by Using TGA Technique. AIP Conf. Proc..

[59] Sorolla-Rosario D., Llorca-Porcel J., Pérez-Martínez M., Lozano-Castelló D., Bueno-López A. (2022). Study of Microplastics with Semicrystalline and Amorphous Structure Identification by TGA and DSC. J. Environ. Chem. Eng..

[60] Tarani E., Arvanitidis I., Christofilos D., Bikiaris D.N., Chrissafis K., Vourlias G. (2023). Calculation of the Degree of Crystallinity of HDPE/GNPs Nanocomposites by Using Various Experimental Techniques: A Comparative Study. J. Mater. Sci..

[61] van der Wal A., Mulder J., Gaymans R. (1998). Fracture of Polypropylene. Polymers.

[62] Chi X., Cheng L., Liu W., Zhang X., Li S. (2018). Characterization of Polypropylene Modified by Blending Elastomer and Nano-Silica. Materials.

[63] Mohagheghian I., McShane G.J., Stronge W.J. (2015). Impact Perforation of Monolithic Polyethylene Plates: Projectile Nose Shape Dependence. Int. J. Impact Eng..

[64] Lomovskoy V.A., Shatokhina S.A. (2024). Relaxation Phenomena in Low-Density and High-Density Polyethylene. Polymers.

[65] Djoković V., Kostoski D., Dramićanin M.D., Suljovrujić E. (1999). Stress Relaxation in High Density Polyethylene. Effects of Orientation and Gamma Radiation. Polym. J..

[66] Jahandideh M., Sararoudi S.S., Barangi L. (2016). Stress Relaxation Behavior of Polyolefin Polymer Blends Based on PP/HDPE. AIP Conf. Proc..

[67] Mainardi F. (2020). Why the Mittag-Leffler Function Can Be Considered the Queen Function of the Fractional Calculus?. Entropy.

[68] Ryapolov P.A., Postnikov E.B. (2021). Mittag–Leffler Function as an Approximant to the Concentrated Ferrofluid’s Magnetization Curve. Fractal Fract..

[69] Imoisili P.E., Makhatha M.E., Jen T.-C. (2024). Artificial Intelligence Prediction and Optimization of the Mechanical Strength of Modified Natural Fibre/MWCNT Polymer Nanocomposite. J. Sci. Adv. Mater. Devices.

[70] Nouri Y., Ghanbari M.A., Fakharian P. (2024). An Integrated Optimization and ANOVA Approach for Reinforcing Concrete Beams with Glass Fiber Polymer. Decis. Anal. J..

[71] Owolabi R.U., Usman M.A., Kehinde A.J. (2018). Modelling and Optimization of Process Variables for the Solution Polymerization of Styrene Using Response Surface Methodology. J. King Saud Univ. Eng. Sci..

[72] Maurya A., Kumar P., Sinha S. (2024). Optimization of Nanofiller Compositions for Enhancing Thermo-Mechanical Properties of Epoxy-Based Composites through the Application of Response Surface Methodology with Central Composite Design. J. Indian Chem. Soc..

[73] Ramanathan G., Hassan M., Rochev Y. (2024). Optimising the Viscoelastic Properties of Hyaluronic Acid Hydrogels through Colloidal Particle Interactions: A Response Surface Methodology Approach. Colloids Surf. A Physicochem. Eng. Asp..

